# Prevalence and diversity of *Rickettsia* species in ectoparasites collected from small rodents in Lithuania

**DOI:** 10.1186/s13071-018-2947-9

**Published:** 2018-06-28

**Authors:** Jana Radzijevskaja, Evelina Kaminskienė, Indrė Lipatova, Dalytė Mardosaitė-Busaitienė, Linas Balčiauskas, Michal Stanko, Algimantas Paulauskas

**Affiliations:** 1 Faculty of Natural Sciences, Vytautas Magnus University, Vileikos 8, LT-44404 Kaunas, Lithuania; 2 Laboratory of Mammalian Ecology, Nature Research Centre, Akademijos st. 2, LT-08412 Vilnius, Lithuania; 3 Institute of Parasitology and Institute of Zoology, Slovak Academy of Sciences, Hlinkova 3, 04000 Košice, Slovakia

**Keywords:** Rickettsiae, *Ixodes ricinus*, Laelapidae mites, Siphonaptera fleas, Rodents, Lithuania

## Abstract

**Background:**

Rickettsiae are emerging pathogens causing public health problems in many countries around the world. *Rickettsia* spp. are found in association with a wide range of arthropods which feed on different species of animals. However, the distribution and natural cycle of *Rickettsia* species and their association with different arthropod vectors are not fully established. The aim of this study was to investigate the presence and prevalence of *Rickettsia* spp. in ticks, mites and fleas parasitizing different species of small mammals in Lithuania and to molecularly characterize the *Rickettsia* spp. obtained from different ectoparasites.

**Results:**

A total of 1261 ectoparasites (596 *Ixodes ricinus* ticks, 550 mites of five species and 115 fleas of eight species) collected from 238 rodents in Lithuania during 2013–2014 were investigated for the presence of *Rickettsia* pathogens. Infection rates were calculated as the maximum likelihood estimation (MLE) with 95% confidence intervals (CI). The infection rate varied among ectoparasites and was found highest in fleas 43.5%, followed by *I. ricinus* ticks (MLE = 26.5%; 95% CI: 22.2–31.3%) and then mites (MLE = 9.3%; 95% CI: 7.0–12.2%). Sequence analysis of partial *gltA* and *17kDa* genes revealed the presence of *Rickettsia helvetica*, *R. felis*, *R. monacensis*, *Rickettsia* sp*.* and rickettsial endosymbionts. Four *Rickettsia* spp. were identified in fleas, while three *Rickettsia* spp. were identified in Laelapidae mites and only one (*R. helvetica*) in *I. ricinus* ticks.

**Conclusions:**

To our knowledge, this is the first report of the occurrence and molecular characterization of *Rickettsia* spp. in 11 species of ectoparasites of small rodents in Lithuania. The present data extend the knowledge on the distribution of *Rickettsia* spp. and their association with different arthropod vectors. Prior to our study, *R. felis* had never been identified in Lithuania. To our knowledge, this is also the first report of *R. felis* in *L. agilis* and *H. microti* mites and in *Ct. agyrtes* and *H. talpae* fleas, as well as the first detection of *R. monacensis* in *Ct. agyrtes* fleas.

**Electronic supplementary material:**

The online version of this article (10.1186/s13071-018-2947-9) contains supplementary material, which is available to authorized users.

## Background

Rickettsiae are obligate intracellular Gram-negative bacteria from the order Rickettsiales that are characterised by complex life-cycles and a diversity of hosts and transmission strategies. Hosts of *Rickettsia* spp. are found in freshwater, marine and terrestrial habitats, and include protozoans, arthropods, vertebrates, photosynthetic algae and plants [1–3].

*Rickettsia* spp. are best known as human pathogens vectored mainly by hematophagous arthropods and causing public health problems in many countries around the world. Some species can cause diseases in other mammals and birds. *Rickettsia* species are horizontally transmitted to vertebrates by a variety of arthropod vectors which feed on different species of animals. To date, ticks (Ixodidae), lice (Phtiraptera) and fleas (Siphonaptera) are known to be competent vectors of rickettsial agents. However, most *Rickettsia* spp. have been found exclusively in non-hematophagous arthropods with no known secondary host [3].

Currently, the genus *Rickettsia* comprises 31 recognized species and numerous uncharacterized strains causing diseases in both humans and domestic and wild animals [4–6]. *Rickettsia* species and associated human clinical diseases vary depending on the geographical location [1, 4]. The genus *Rickettsia* was traditionally classified into three groups based on phenotypic characters: the spotted fever group (SFG), the typhus group (TG) and the scrub typhus group (STG). The development of molecular tools over recent decades has resulted in reorganizations in the rickettsiae taxonomy. Phylogenomic studies have shown that the genus could be divided into four different phylogenetic groups: (i) the SFG associated mainly with ticks and, less frequently, with fleas and mites, consisting of 23 validated species (using a whole genome approach, *Rickettsia helvetica* was characterized as separate group from SFG); (ii) the typhus group which includes the agents of epidemic typhus and murine typhus associated with lice and fleas; (iii) an ancestral group (consisting of *R. bellii* and *R. canadensis*); and (iv) a transitional group whose members are *R. akari* and *R. felis* [7].

In Europe, rickettsioses are caused mainly by tick-borne SFG *Rickettsia* [1, 2, 4]. Ticks play important roles as vectors and sometimes as reservoirs in the ecology of these pathogens. The tick *Ixodes ricinus* is the main vector of *R. helvetica* and *R. monacensis*, while *R. massiliae*, *R. raoultii*, *R. vini* and other rickettsial species that have yet to be fully characterized have also been detected in this tick species [1, 8–11].

Mites of the family Laelapidae (Acari: Mesostigmata) are distributed worldwide and are the most common ectoparasites of small rodents. Parasitic species from this family are frequently found on the bodies of rodents or in their nests [12–15]. The role of mesostigmatid mites in the circulation of some disease agents has been previously confirmed and recent studies have also demonstrated that mesostigmatid mites may be reservoirs as well as vectors of some pathogenic rickettsiae (reviewed by Merhej et al. [4], Miťková et al. [12]). Fleas from the order Siphonaptera are another group of blood-sucking ectoparasites found on rodents [16]. Fleas are important vectors for various pathogens including viruses, bacteria and tapeworms. Different *Rickettsia* species have been detected in more than 15 flea species [4]. In Europe, fleas infesting small rodents have been found to be infected with *R. felis*, *R. helvetica* and rickettsial endosymbionts [17, 18].

Information on the circulation of rickettsiae in the territories of the Baltic countries is scarce. In Lithuania, the presence and prevalence of two SFG rickettsiae has been previously reported in questing ixodid ticks [19]: *R. helvetica* has been identified in *I. ricinus* (with a prevalence of 17%) and *R. raoultii* in *Dermacentor reticulatus* (4.9%). So far, no reported human clinical cases due to infection by *Rickettsia* species have been registered in Lithuania. Detection of *R. helvetica*, *R. monacensis* and “*Candidatus* R. tarasevichiae” have been reported in questing *I. ricinus* and *I. persulcatus* ticks in Estonia [20]. In Latvia, *R. helvetica* has been identified in *I. ricinus* ticks from birds [21]. There are currently no published reports of the presence of *Rickettsia* spp. in mites and fleas in Lithuania, nor in other Baltic states.

The distribution and natural cycle of *Rickettsia* spp. and their association with different arthropod vectors are not fully established. In order to extend knowledge on the relationships between arthropod vectors, hosts and *Rickettsia* pathogens, we aimed to investigate the presence and prevalence of *Rickettsia* spp. in ticks, mites and fleas parasitizing different species of small rodents in Lithuania and to molecularly characterize the *Rickettsia* spp. obtained from different ectoparasites.

## Methods

### Sample collection

**Fig. 1:**
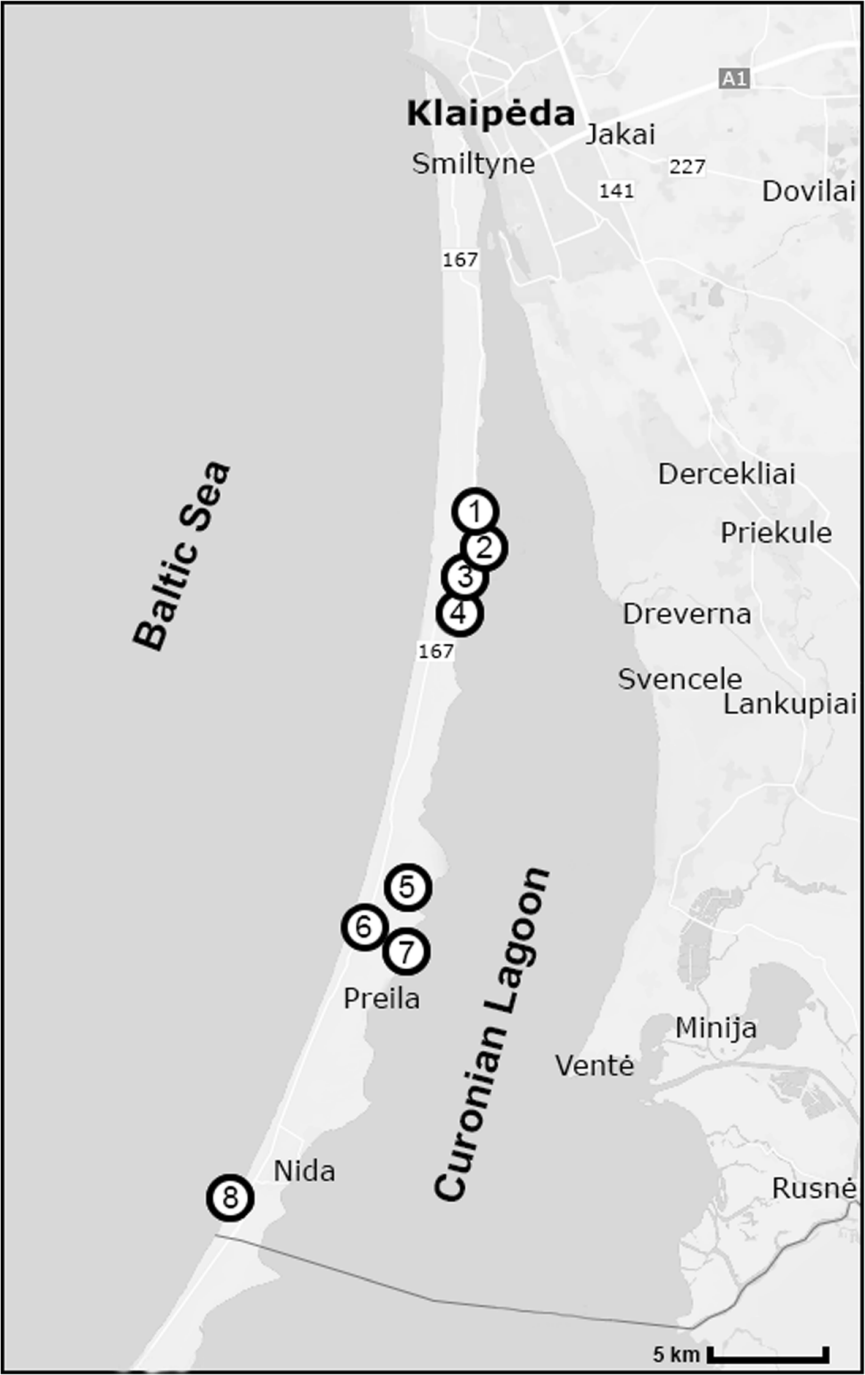
Small rodents trapping sites in Curonian Spit, Lithuania: 1, Amber Gulf; 2, Juodkrante; 3, Grobstas Cape; 4, Great Cormorant and Grey Heron colony; 5, Pervalka Gulf; 6, Nida Dump; 7, Karvaiciai Gulf; 8, Lybis Cape

Trapping of rodents was conducted on the Curonian Spit of Lithuania during 2013–2014. The Curonian Spit is a narrow sand peninsula (2 km wide and 98 km long, with half of the length in Lithuania) in the southeastern part of the Baltic Sea separating the Curonian Lagoon from the Baltic Sea [22]. The climate of the Curonian Spit is intermediate between marine and continental and is characterized by frequent and intense variability of weather, relatively mild winters and temperately warm summers. According to the phytogeographical classification, the territory of the Curonian Spit is considered to be within the zone of mixed coniferous-broad-leaved forest. The territory of the Curonian Spit is characterized by considerable flora species diversity [22]. Small rodents were captured at eight sampling sites (Fig. 1) located in different habitats (coastal meadow, mixed forest and forest-meadow ecotone). The names and coordinates of the sampling sites are provided in Additional file 1: Table S1.

Rodents were captured with live-traps and/or snap traps. All trapped rodents were marked and identified by species and sex. Altogether, 238 small rodents representing six species were collected: *Apodemus flavicollis* (*n* = 167), *Myodes glareolus* (*n* = 23), *Micromys minutus* (*n* = 37), *Microtus oeconomus* (*n* = 7), *M. agrestis* (*n* = 2) and *M. arvalis* (*n* = 2).

Ectoparasites were collected from hosts using tweezers and placed into 1.5 ml tubes with 70% ethanol. The collected ticks, mites and fleas were identified by the use of taxonomic keys [13, 16, 23–25].

### DNA extraction

DNA from ticks, mites and fleas was extracted by using 2.5% ammonium hydroxide solution [26]. DNA from engorged ticks was extracted using a Genomic DNA Purification Kit (Thermo Fisher Scientific, Vilnius, Lithuania) according to the protocol suggested by the manufacturer. Ectoparasites were processed individually or pooled (2–10 specimens) in groups from each host by species, life stage, sex and location.

### PCR amplification and DNA sequencing

*Rickettsia* DNA in ectoparasites was detected using a nested PCR that targeted partial *gltA* gene (encoding rickettsial citrate synthase of *Rickettsia* spp.) and two primer sets RpCS.877p/RpCS.1258n (external primers) and RpCS.896p/RpCS.1233n (internal primers) as previously described [12]. The obtained specific products of 338 base pairs were considered as a positive result.

All *gltA*-positive samples for *Rickettsia* were further examined using a semi-nested PCR which amplifies the 450 bp fragment of the *17-kDa* gene (encoding rickettsiae genus-specific 17-kDa outer membrane antigen) using the primers Rr17k.1p/Rr17k.539n and Rr17k.90p as described [27]. A negative control (double-distilled water) and a positive control (DNA of *Rickettsia*-infected ticks, confirmed by sequencing) were included in every PCR run. PCR products were subjected to electrophoresis on 1.5% agarose gel and analyzed by UV transilluminator.

To prevent cross-contamination, separate rooms were used for DNA isolation, PCR-mix preparation, the PCR and nested-PCR reactions. Negative controls, which consisted of sterile, double-distilled water added to the first PCR mix rather than DNA, were included after every five experimental samples.

A selected number of *Rickettsia*-positive samples for both genes were purified using the GeneJET™ Gel Extraction Kit (Thermo Fisher Scientific) and sequenced (Macrogen, Amsterdam, the Netherlands). Obtained DNA sequences were edited using the MEGA 6.05 software package [28] and aligned to available data in GenBank with BLASTn. The *gltA* and 17kDa protein-coding gene based phylogenetic trees were constructed using the maximum-likelihood (ML) and neighbor-joining (NJ) methods. The most appropriate model of nucleotide substitution was determined according to the Bayesian information criterion (BIC) using the program jModelTest2 [29, 30].

Partial *17kDa* and *gltA* gene sequences for representative samples were submitted to the GenBank database under the accession numbers MF491767-MF491779, MH454244 and MF491780-MF491791, MH454245- MH454247 respectively.

### Statistical analysis

Infection rates for pooled ticks and mites were calculated using maximum likelihood estimation (MLE) method with 95% confidence intervals (CI) for unequal pool sizes and expressed as the MLE of infection rate per 100 ticks/mites. MLE calculations are based on the number of pools, pool sizes (number of individuals per pool), and the number of positive pools [31]. We used the PooledInfRate estimation software (version 4.0) add-on with Microsoft Excel (http://www.cdc.gov/westnile/resourcepages/mosqSurvSoft.html).

Differences of prevalence of *Rickettsia* spp. in ectoparasites were analysed using a Chi-square test with Statistica for Windows, version 7.0.

## Results

From 238 trapped rodents, 181 (76.1%) were infested with ectoparasites. A total of 1261 ectoparasites of 18 different species were collected from 136 individuals of *A. flavicollis*, 23 of *M. minutus*, 15 of *M. glareolus*, 5 of *M. oeconomus*, one of *M. arvalis* and one of *M. agrestis*. The majority of ectoparasites (*n* = 1135; 90.0%) were removed from *A. flavicollis*.

The infested rodents were parasitized by *I. ricinus* ticks, five species of mites of the family Laelapidae (*Laelaps agilis*, *Hyperlaelaps microti*, *Haemogamassus nidi*, *Eulaelaps stabularis* and *Myonyssus gigas*) and eight species of fleas (*Ctenophthalmus agyrtes*, *Ct. assimilis*, *Hystrichopsylla talpae*, *H. orientalis*, *Megabothris turbidus*, *M. walkeri*, *Palaeopsylla soricis* and *Nosopsyllus fasciatus*).

Altogether, 596 immature *I. ricinus* (8 nymphs, 588 larvae), 550 mites and 115 fleas were collected. All nymphs and 62 larvae of *I. ricinus*, all fleas and 82 mites were examined individually (Tables 1, 2). The remaining 526 *I. ricinus* larvae and 468 mites (*L. agilis*) were grouped into pools of 2 to 10 individuals (29 × 2, 14 × 3, 24 × 4, 24 × 5, 23 × 6, 1 × 7, 7 × 8 and 1 × 9 for the *I. ricinus* larvae; 31 × 2, 17 × 3, 8 × 4, 11 × 5, 8 × 6, 7 × 7, 11 × 8, 7 × 9 and 2 × 10 for the mites) (Table 2). Larvae of ticks and mites from different rodents were not mixed in the same pools. A total of 259 individual ectoparasites (ticks, mites, fleas) and 225 pools, representing 526 *I. ricinus* ticks and 468 mites, were screened for the presence of rickettsial DNA by PCR targeting the *gltA* gene (Tables 1, 2).

**Table 1:** Presence of *Rickettsia* spp. in fleas collected from different species of small rodents on the Curonian Split, Lithuania

Rodent species	Fleas infected by *Rickettsia* spp., n/N												Total
	Ct. ag		Ct. as	H. t		H. o	M. t		M. w		P. s	N. f	
	**♀**	**♂**	**♂**	**♀**	**♂**	**♂**	**♀**	**♂**	**♀**	**♂**	**♀**	**♂**	
*A. fla*	14/30	10/25	1/1	0/2	1/1	–	5/16	2/8	3/3	2/2	-	0/1	38/89
*M. min*	0/4	0/2	–	–	1/2	0/1	1/1	1/1	–	–	–	–	3/11
*M. gla*	–	–	–	0/1	–	–	1/3	1/1	–	1/1	–	–	3/6
*M. agr*	–	–	–	–	–	–	–	–	1/1	–	–	–	1/1
*M. oec*	2/3	–	–	–	–	–	–	1/1	1/2	0/1	1/1	–	5/8
Total	16/37	10/27	1/1	0/3	2/3	0/1	7/20	5/11	5/6	3/4	1/1	0/1	50/115

*Abbreviations*: N, number of tested fleas; n, number of infected fleas; ♀, female; ♂, male; Ct. ag,* Ctenophthalmus agyrtes*; Ct. as, *Ctenophthalmus assimilis*; H. t, *Hystrichopsylla talpae*; H. o, *Hystrichopsylla orientalis*; M. t, *Megabothris turbidus*; M. w, *Megabothris walkeri*; P. s, *Palaeopsylla soricis*; N. f, *Nosopsyllus fasciatus*; A. fla, *Apodemus flavicollis*; M. min, *Micromys minutus*; M. gla, *Myodes glareolus*; M. arv, *Microtus arvalis*; M. agr, *Microtus agrestis*; M. oec, *Microtus oeconomus*

**Table 2:** Presence of *Rickettsia* spp. in *Ixodes ricinus* ticks and Laelapidae mites collected from different species of small rodents on the Curonian Spit, Lithuania

Rodent species	*I. r*		*L. a*		H. m	M. g		E. s	Hg. n
	Larvae no. positive pools/No. pools (no. ticks in pools); single n/ N; (total pools/total ticks tested); % MLE (95% CI)	Nymphs n/ N	no. positive pools /No. pools (no. mites in pools); single n/N; (total pools /total mites tested); % MLE (95% CI)		n/ N	n/ N		n/ N	n/ N
			**♀**	**♂**	**♀**	**♀**	**♂**	**♀**	**♀**
*A. fla*	87/ 115 (502); 12/56; (171/558)	1/3	31/88 (428); 1/20; (108/448)	0/21	0/5	1/2	0/1	2/5	0/3
*M. min*	1/ 2(5); 2/6; (8/11)	1/2	4/8 (19); 2/5; (13/24)	0/1	3/3	0/0	0/0	0/4	0/0
*M. gla*	2/3(8)	2/2	0/5 (19); 0/2; (7/21)	0/1	0/3	0/0	0/0	0/1	0/0
*M. arv*	1/1(6)	0/0	0/0	0/0	0/0	0/0	0/0	0/0	0/0
*M. oec*	1/2(5)	1/1	0/1(2); 0/1; (2/3)	0/2	0/0	0/0	0/0	0/0	0/0
*M. agr*	0/0	0/0	0/0	0/0	0/2	0/0	0/0	0/0	0/0
Total	87/123 (526); 14/62; (185/588); 25.7% (21.4–30.5)	5/8	35/102 (468); 3/28; (130/496); 9.0% (6.6–12.1)	0/25	3/13	1/2	0/1	2/10	0/3

*Abbreviations*: *N*, number of ticks/mites tested; *n*, number infected; MLE, maximum likelihood estimates; ♀, female; ♂, male; *I. r*, *Ixodes ricinus*; *L. a*, *Laelaps agilis*; *H. m*, *Hyperlaelaps microti*; *M. g*, *Myonyssus gigas*; *E. s*, *Eulaelaps stabularis*; *Hg. n*, *Haemogamasus nidi*; *A. fla*, *Apodemus flavicollis*; *M. min*, *Micromys minutus*; *M. gla*, *Myodes glareolus*; *M. arv*, *Microtus arvalis*; *M. oec*, *Microtus oeconomus*; *M. agr*, *Microtus agrestis*

Combined data including both PCR-positive ectoparasites tested individually and in pools showed that at least 20.4% (95% CI: 18.0–23.0%) of ectoparasites harbored rickettsiae. DNA of *Rickettsia* spp. was identified in 22.6 % (14 of 62) of individually tested *I. ricinus* larvae, in 5 out of 8 nymphs and in 70.7% of 123 larvae pools. In the case of mites, 11.0% (9 out of 82) of single specimens and 34.3% of 102 pools (*L. agilis*) tested positive (Table 2).

Among the examined ectoparasites, the highest infection rate was found in fleas (43.5%; *χ*^2^ = 17.62, *P* < 0.0001; *χ*^2^ = 63.19; *P* < 0.0001), followed by *I. ricinus* ticks (MLE = 26.5%, 95% CI: 22.2–31.3%) and then mites (MLE = 9.3%, 95% CI: 7.0–12.2%).

*Rickettsia* DNA was detected in eleven species of ectoparasites. *Rickettsia*-infected fleas were found parasitizing five rodent species, while infected *I. ricinus* ticks and mites were found only on two species of rodents (Table 1, 2).

*Rickettsia* spp. were detected in six flea species collected from *A. flavicollis*, *M. glareolus*, *M. minutus*, *M. oeconomus* and *M. agrestis* rodents (Table 1). The most prevalent species was *Ct. agyrtes* (55.7%, *n* = 64), followed by *M. turbidus* (27%, *n* = 31), and the prevalence of *Rickettsia* spp. in these ectoparasites was 40.63% (26/64) and 38.7% (12/31), respectively (Table 1). A total of 42.7% of fleas collected from *A. flavicollis* and 27.3% from *M. minutus* harboured *Rickettsia* pathogens.

*Rickettsia*-positive *I. ricinus* ticks were found on *A. flavicollis*, *M. glareolus*, *M. minutus*, *M. oeconomus* and *M. arvalis* rodents. Nearly 94.1% of *I. ricinus* ticks analyzed were collected from *A. flavicollis*, and the MLE of *Rickettsia* infection in *I. ricinus* parasitizing these mice was 26.9% (95% CI: 22.4–31.9%). The overall MLE of the infection rate in *I. ricinus* larvae was 25.7% (95% CI: 21.4–30.5%) (Table 2).

*Rickettsia* DNA was detected in four mite species collected from *A. flavicollis* and *M. minutus* rodents. *Rickettsia* spp. were detected only in females of mites (Table 2). The dominant species of mite found on small rodents was *L. agilis* (521/550). The overall MLE of *Rickettsia* for *L. agilis* was 8.5% (95% CI: 6.2–11.4%), with the MLE in *L. agilis* females being 9.0% (95% CI: 6.6–12.1%) (Table 2). *Rickettsia* DNA was also detected in *H. microti* (3/13, 23.08%) from *M. minutus* and in *E. stabularis* (2/10, 20.0%) and *M. gigas* (1/3) from *A. flavicollis* (Table 2).

**Fig. 2:**
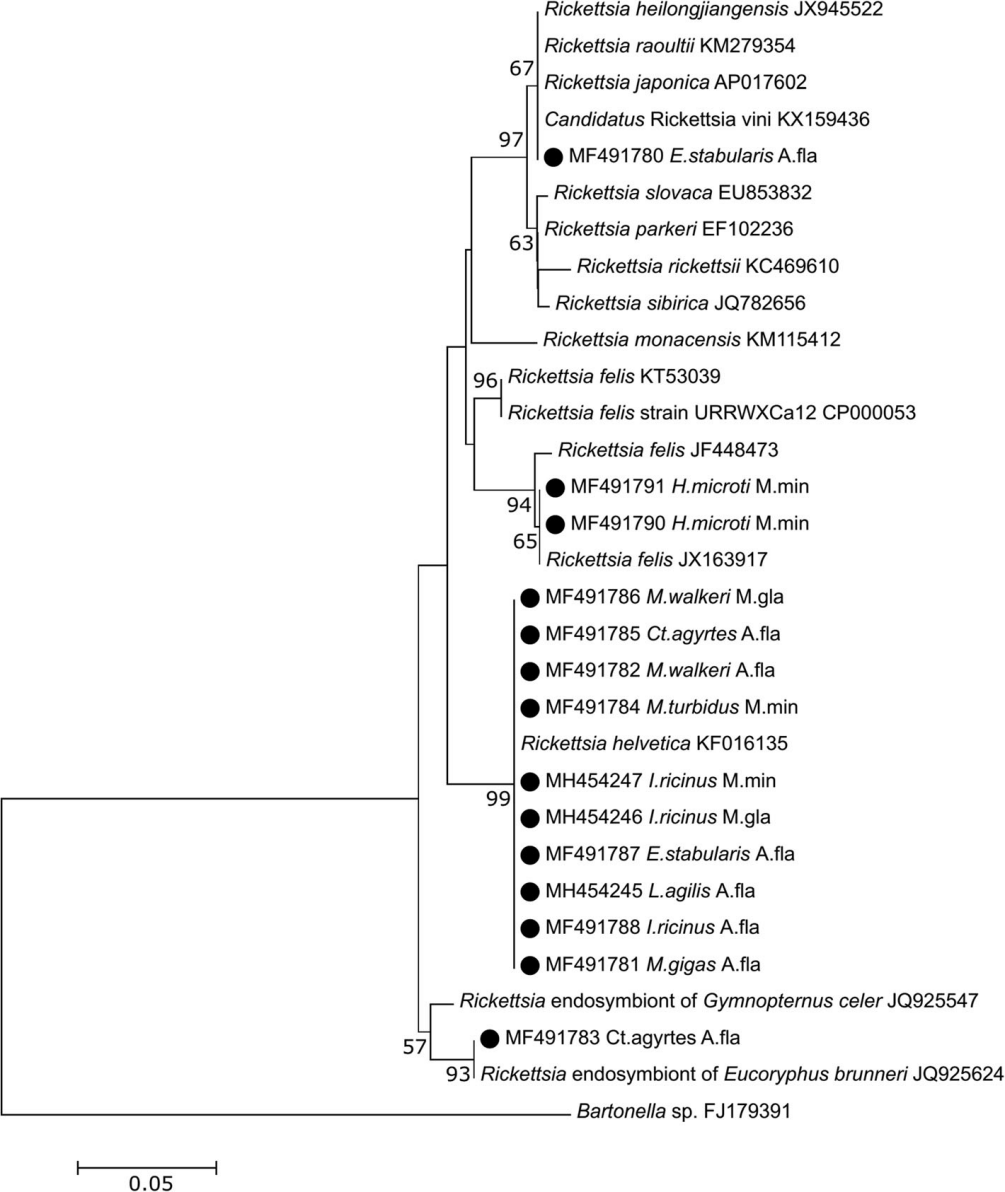
Neighbour-joining phylogenetic tree for the partial *gltA* gene of *Rickettsia* spp. The phylogenetic tree was created using the Kimura 2-parameter model with a discrete Gamma-distribution (+G) and bootstrap analysis of 1000 replicates. Identification source (host) is given after the names of species. Samples sequenced in the present study are marked. Sequence MF491788 is representative of six other samples sequenced in this study (all derived from *I. ricinus* obtained on *A. flavicolis*); Sequence MH454245 is representative of two other samples sequenced in this study (derived from *L. agilis* obtained on *A. flavicolis*). *Abbreviations*: A. fla, *Apodemus flavicollis*; M. min, *Micromys minutus*; M. gla, *Myodes glareolus*

*Rickettsia*-positive PCR products of good*-*quality were subjected to sequence analysis. A total of 44 sequences of *gltA* (*n* = 22) and *17kDa* (*n* = 22) genes were analyzed, derived from *I. ricinus* ticks, four species of mites and four species of fleas collected from *A. flavicollis*, *M. minutus* and *M. glareolus* rodents. Sequence analysis of the partial *gltA* gene revealed the presence of four different species of *Rickettsia* in the analysed samples: (i) *R. helvetica*: sequences derived from the tick *I. ricinus*, mites *L. agilis*, *E. stabularis* and *M. gigas* and fleas *Ct. agyrtes*, *M. turbidus* and *M. walkeri* were 100% identical to each other and to the *R. helvetica* sequences deposited in GenBank (KF016135); (ii) *R. felis*: sequences derived from two *H. microti* mites were 97% similar (nucleotide sequences differed at 9 positions) to a validated bacterium *R. felis* (CP000053), 99% similar to *R. felis* detected in *A. agrarius* from South Korea (GenBank: JF448473) and shared 100% identify with *R. felis* which was detected in *E. stabularis* mites from China (GenBank: JX163917); (iii) *Rickettsia* sp.: the sequence isolated from *E. stabularis* was 100% identical to the closely phylogenetically related sequences deposited in GenBank for *Rickettsia vini* (GenBank: KX159436), *R. japonica* (GenBank: AP017602), *R. heilongjiangensis* (GenBank: JX945522) and *R. raoultii* (GenBank: KM279354); (iv) the *gltA* sequence isolated from the *Ct. agyrtes* flea was 99–100% identical to the *Rickettsia* endosymbiont of *Eucoryphus brunneri* (family Dolichopodidae, order Diptera; GenBank: JQ925624) and the *Rickettsia* endosymbiont of *Gymnopternus celer* (family Dolichopodidae, order Diptera; GenBank: JQ925547) (Fig. 2).

**Fig. 3:**
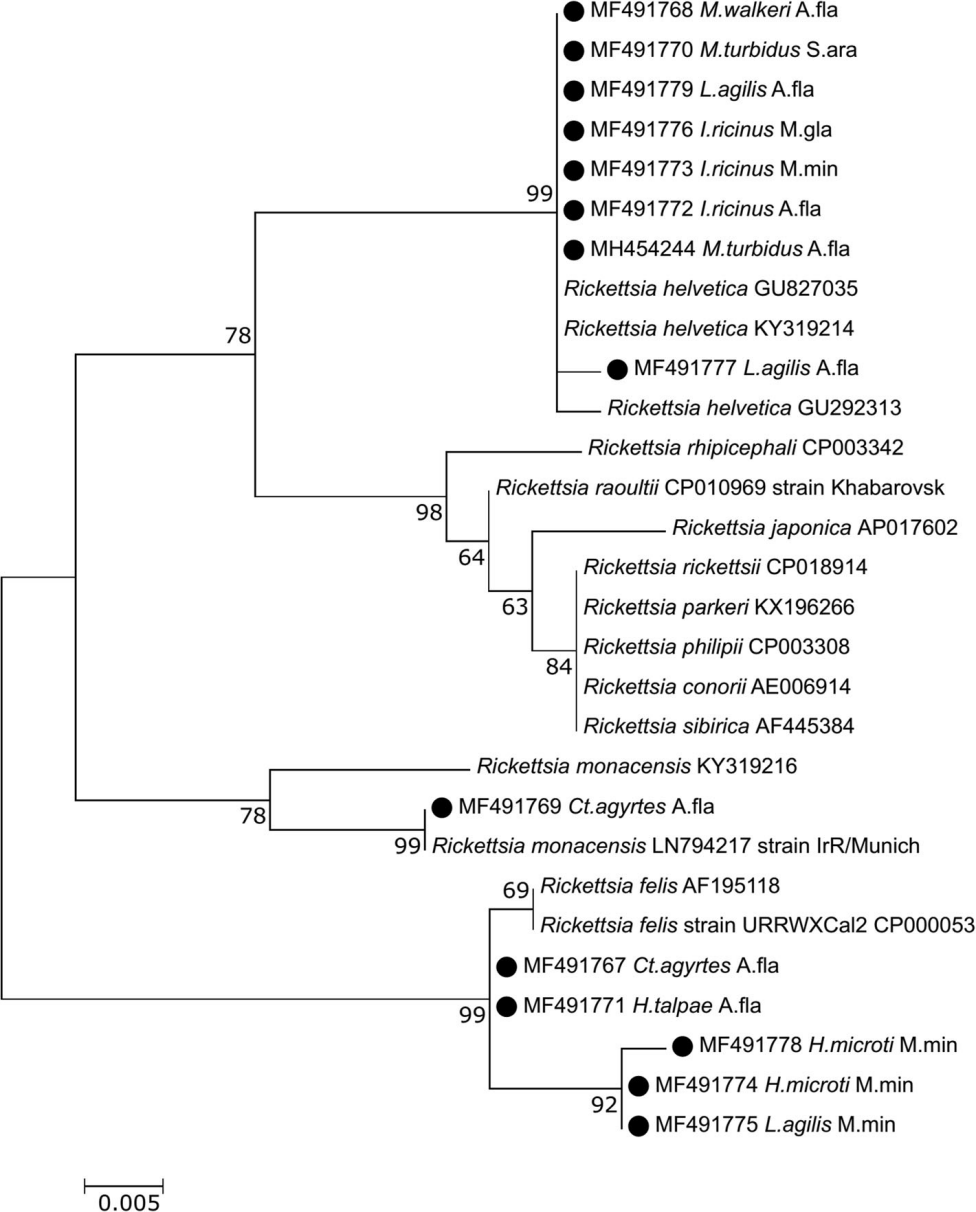
Maximum-likelihood phylogenetic tree for the partial *17kDa* gene of *Rickettsia* spp. The phylogenetic tree was created using the Tamura-Nei model with a discrete Gamma-distribution (+G) and bootstrap analysis of 1000 replicates. Identification source (host) and is given after the names of species. Samples sequenced in the present study are marked. Sequence MF491772 is representative of six other samples sequenced in this study (all derived from *I. ricinus* of *A. flavicolis*). Sequence MF491779 is representative of two other samples sequenced in this study (derived from *L. agilis* of *A. flavicolis*). *Abbreviations*: A. fla – *Apodemus flavicollis*; M. min – *Micromys minutus*; M. gla – *Myodes glareolus*

PCR and sequence analysis of the partial *17kDa* gene revealed the presence of three *Rickettsia* species: (i) *R. helvetica*: isolates derived from *I. ricinus* ticks, *L. agilis* mites and two species of fleas *M. walkeri* and *M. turbidus* were 99–100% identical (with one nucleotide difference) to the *R. helvetica* isolates deposited in GenBank (GU827035, KY319214, GU292313); (ii) *R. felis* was detected in four species of ectoparasites: *H. microti* and *L. agilis* mites and *Ct. agyrtes* and *H. talpae* fleas. The amplified fragments of the *17kDa* gene of rickettsiae in the *Ct. agyrtes* and *H. talpae* samples were 100% identical to each other and 99% similar (differed in one nucleotide) to a validated bacterium *R. felis* (GenBank: CP000053), while sequences isolated from *H. microti* and *L. agilis* were 99% identical to each other and differed in four to five nucleotide positions from *R. felis* (GenBank: CP000053); (iii) *R. monacensis*: the *17kDa* gene sequence derived from the *Ct. agyrtes* flea was 99–100% identical to the *R. monacensis* sequences deposited in GenBank (GU292312, LN794217) (Fig. 3).

## Discussion

In this study, we report the occurrence and the molecular characterization of *Rickettsia* spp. in eleven species of ectoparasites parasitizing small rodents on the Curonian Spit, West Lithuania. The frequency of *Rickettsia* spp. infections in rodent-derived ectoparasites varied among species and was found highest in fleas, followed by *I. ricinus* ticks and then mites.

Small rodents are important hosts for the immature stages of ixodid ticks and are considered carriers and reservoir hosts of tick-borne pathogens such as tick-borne encephalitis virus [32], *Borrelia burdorferi* (*s.l.*) [33], *Borrelia miyamotoi* [34], *Babesia microti* [35], *Anaplasma phagocytophilum* and “*Candidatus* Neoehrlichia mikurensis” [36]. The reservoir role of wild rodents in rickettsiae life-cycles is still unclear and there are a lack of studies focusing on the investigation of rickettsial pathogens in rodents and their ectoparasites across Europe [12, 17, 37, 38]. Several molecular studies conducted in the Netherlands, Germany, Slovakia, Poland and Austria have showed the presence of rickettsial DNA in European rodents with a prevalence ranging from 2.7 to 29%. Infected rodents more frequently harbored *R. helvetica* than other rickettsiae species [12, 18, 37, 39, 40].

The presence of rickettsiae in ectoparasites may result from the acquisition of bacteria *via* blood meals from rickettsiemic rodents. Some SFG rickettsiae are thought to circulate in enzootic or epizootic cycles between wild vertebrates and arthropod vectors [41]. The high prevalence of *R. helvetica* previously obtained in small rodents suggests that they may play an important role as potential natural reservoir hosts for this pathogen [37, 39, 42]. In the present study, the most frequently captured rodent species in all habitats was *A. flavicollis*, followed by *M. minutus* and *M. glareolus* (captured in grasslands). *Rickettsia* spp. harboured only rodents of three species *A. flavicollis* 32.8%, *M. minutus* 45.9% and *M. glareolus* 14.3%, while other rodent species were found to be non-rickettsiemic (data not shown) [42]. All infected rodents harboured *R. helvetica* [42]. Our data showed that 66.6% of *Rickettsia*-infected *I. ricinus* ticks, 59% of Laelapidae mites and 55% of fleas were derived from rodents which had been previously proved to be non-rickettsiemic, while 33.4 % of *Rickettsia*-infected *I. ricinus* ticks, 41% of Laelapidae mites and 45% of fleas were collected from infected rodents [42]. This could have potentially influenced the detection rates estimated in our study for ectoparasites that harboured *R. helvetica*. It seems that PCR-positive ticks, mites and fleas feeding on non-infected rodents, as well as ectoparasites which harboured other *Rickettsia* species, did not acquire these pathogens through blood meals on rodents. Mites and fleas in these cases could be infected during previously feedings on another hosts (e.g. in common nests). Occurrence of PCR-positive *I. ricinus* larvae on non-infected rodents probably show transovarial transmission from engorged females.

To our knowledge, the present study is the first report on the presence of *Rickettsia* spp. in fleas and mites from the Baltic countries. Fleas and mites feeding on rodents harboured multiple *Rickettsia* species, including *R. helvetica*, *R. felis*, *R. monacensis*, rickettsial endosymbiont and *Rickettsia* sp. Four *Rickettsia* spp. were identified in fleas, while three *Rickettsia* spp. were identified in Laelapidae mites. In *Rickettsia*-positive *I. ricinus* samples subjected for sequence analysis, only *R. helvetica* species was detected.

*Rickettsia helvetica* is a human pathogenic *Rickettsia* species which was first discovered in *I. ricinus* from Switzerland in 1979 [43]. Human cases have been reported from Sweden, France, Switzerland, Italy, Denmark, Austria and Slovakia (reviewed by Parola et al. [1]). *Ixodes ricinus* is considered to be the main vector and a natural reservoir of *R. helvetica* [44] due to the ability of rickettsiae to survive perpetually in ticks and to be transmitted transstadially and transovarially [1]. To date, *R. helvetica* has been detected in *I. ricinus* in many European countries with a highly variable prevalence. This species has been previously detected in questing *I. ricinus* ticks in Lithuania with a prevalence ranging in different locations between 0–31.3% [19].

*Rickettsia helvetica* has also been isolated from other tick species such as *D. reticulatus*, *I. hexagonus* and *I. arboricola*, as well as from mites and fleas [1]. This species has been detected in *Ct. agyrtes* males collected from *A. agrarius* in Slovakia [17] and in two out of 24 fleas isolated from *M. glareolus* and *A. sylvaticus* in the Netherlands [39]. Miťková et al. [12] reported for the first time the detection of *R. helvetica* in *L. agilis* and *Hg. nidi* mites infesting small rodents in Slovakia.

In our study, *R. helvetica* was detected in *I. ricinus* ticks, *L. agilis* and *M. gigas* mites and *Ct. agyrtes*, *M. turbidus* and *M. walkeri* fleas from *A. flavicollis*; *I. ricinus* ticks and *M. walkeri* fleas from *M. glareolus*, and *M. turbidus* fleas from *M. minutus.* To our knowledge, this is the first detection of *R. helvetica* in *M. gigas* mites and *M. turbidus* and *M. walkeri* fleas. The presence of *R. helvetica* in the immature ticks may result from acquisition pathogens through a blood meal from rickettsiemic rodents or through a vertical (transovarial and transstadial) transmission. Mites and fleas infesting rodents could acquire *R. helvetica* infection while taking a blood meal on a *R. helvetica*-infected rodent or, possibly, by co-feeding with infected *I. ricinus* ticks. Recently, horizontal transmission through a shared blood meal was demonstrated for some rickettsial pathogens [45].

This study is the first report of *R. felis* infection in *Ct. agyrtes* and *H. talpae* fleas collected from *A. flavicollis*, and in *H. microti* and *L. agilis* mites collected from *M. minutus* in Lithuania. *Rickettsia felis* is the causative agent of flea-borne spotted fever in humans [46]. This species has worldwide distribution and infections of *R. felis* have been reported in over 25 countries spanning five continents [47]. The cat flea, *Ctenocephalides felis*, is a main vector and a reservoir of this pathogen [48]. However, more than 24 species of ectoparasites, such as fleas, ticks, mites, lice and mosquitoes, have been demonstrated as vectors for *R. felis* all over the world [4, 13, 17, 27, 49]. Recently, both intra- and interspecific transmission of *R. felis* between co-feeding arthropods on a vertebrate host has been demonstrated [44]. The finding of *R. felis* in naturally infected mites is not unexpected because it has been previously reported in other Mesostigmata mite species collected from rodents in Asia [50, 51]. Sequence analyses of *R. felis* strains isolated from different arthropods revealed genomic heterogeneity and provides evidence for host-specific strain variation [52]. Additionally, increasing numbers of identified *R. felis*-like organisms (RFLOs) in different arthropods have been reported within the last decade.

To our knowledge, this study provides the first evidence of the presence of *R. monacensis* in *Ct. agyrtes* fleas collected from *A. flavicollis* (Fig. 2). *Rickettsia monacensis* is an etiological agent of human rickettsioses distributed throughout Europe. *Ixodes ricinus* is recognized as the main vector of this pathogen [4, 13, 53]. However, not all possible vector species are currently known [53]. *Rickettsia monacensis* has been detected in questing *I. ricinus* ticks in many European countries [1] and has also been detected in immature *I. ricinus* ticks collected from migratory birds in the Russian part of the Curonian Spit [54]. In Lithuania, *R. monacensis* has been previously detected in *I. ricinus* ticks removed from raccoon dogs (GenBank: KT033401). The first occurrence of mites infected with *R. monacensis* has recently been reported in Slovakia [12].

Based on sequence analysis of the *gltA* gene, the *Rickettsia* sp. detected in our study in the mite *E. stabularis* was not identified to the species level. The obtained 268 bp *gltA* sequence showed 100% identity to the corresponding sequences of *R. vini*, *R. japonica*, *R. heilongjiangensis* and *R. raoultii* deposited in the GenBank database. Unfortunately, the fragment of the *17kDa* gene of this sample was not successfully amplified. *R. vini* was first detected in the ticks *I. arboricola* and *I. ricinus* collected from birds in Spain and named “*Candidatus* R. vini” [8, 55]. This bacterium has since been detected in ticks feeding on birds in other European counties and Turkey [56]. The pathogenicity for humans and animals of this *Rickettsia* remains unknown [57]. *Rickettsia vini* has been confirmed in Lithuania in *I. lividus* ticks from sand martin (*Riparia riparia*) nests (GenBank: MH454248).

The present study indicates the presence of phylogenetically distinct rickettsiae in multiple ectoparasites feeding on different rodent hosts. Future studies should be performed to determine the specific roles of different species of mites and fleas parasitizing small rodents in the acquisition and transmission of different *Rickettsia* species.

## Conclusions

To our knowledge, this is the first report of the occurrence and molecular characterization of *Rickettsia* spp. in 11 species of ectoparasites of small rodents in Lithuania. The data presented in this paper extend the knowledge on the distribution of *Rickettsia* species, their association with different arthropod vectors and rodent-parasites interactions. Prior to our study, *R. felis* had never been identified in Lithuania. To our knowledge, this is also the first report of *R. felis* in *L. agilis* and *H. microti* mites and in *Ct. agyrtes* and *H. talpae* fleas, as well as the first detection of *R. monacensis* in *Ct. agyrtes* fleas.

## Additional file

Additional file 1: **Table S1.** Characteristics of sampling sites of rodents in Curonian Spit, Lithuania. (DOCX 14 kb)

## Abbreviations

SFG: Spotted fever group
TG: Typhus group
STG: Scrub typhus group
ML: Maximum-likelihood method
NJ: Neighbor-joining method
BIC: Bayesian information criterion
MIR: Minimum infection rate
