# Supplementary material for: Prevalence and diversity of Rickettsia species in ectoparasites collected from small rodents in Lithuania
Source: Parasit Vectors. 2018 Jun 28;11:375. doi: 10.1186/s13071-018-2947-9 (PMC6025725; doi:10.1186/s13071-018-2947-9)
Supplement: Supplementary file 1 — Table S1. Characteristics of sampling sites of rodents in Curonian Spit, Lithuania. (DOCX 14 kb) [file 13071_2018_2947_MOESM1_ESM.docx]

|  | Site name | Coordinates (Latitude, Longitude) | Habitat |
| --- | --- | --- | --- |
| 1 | Amber Gulf | 55°33'06.0"N, 21°07'31.5"E | coastal meadow |
| 2 | Juodkrante | 55°32'30.9"N, 21°07'02.4"E | ecotone (forest*-*meadow*)* |
| 3 | Grobstas Cape | 55°32'33.6"N, 21°07'13.1"E | coastal meadow |
| 4 | Grey Heron and Cormorant colony | 55°31'08.4"N, 21°06'42.7"E | mixed forest |
| 5 | Pervalka Gulf | 55°24'37.7"N, 21°05'08.4"E | coastal meadow |
| 6 | Nida Dump | 55°23'33.5"N, 21°02'58.4"E | meadow |
| 7 | Karvaiciai Gulf | 55°23'15.4"N, 21°04'19.4"E | coastal meadow |
| 8 | Lybis Cape | 55°16'57.1"N, 20°57'30.8"E | coastal meadow |

**Additional file 1: Table S1.** Characteristics of sampling sites of rodents in Curonian Spit, Lithuania
